# Combinations of isoform-targeted histone deacetylase inhibitors and bryostatin analogues display remarkable potency to activate latent HIV without global T-cell activation

**DOI:** 10.1038/s41598-017-07814-4

**Published:** 2017-08-07

**Authors:** Brice J. Albert, Austin Niu, Rashmi Ramani, Garland R. Marshall, Paul A. Wender, Robert M. Williams, Lee Ratner, Alexander B. Barnes, George B. Kyei

**Affiliations:** 1 Department of Chemistry, Washington University in St. Louis, St. Louis, Missouri 63130 USA; 2 Department of Medicine, Washington University School of Medicine in St. Louis, St. Louis, Missouri 63110 USA; 3 Department of Biochemistry and Molecular Biophysics, Washington University School of Medicine in St. Louis, St. Louis, Missouri 63110 USA; 4 Departments of Chemistry and Chemical and Systems Biology, Stanford University, Stanford California, 94305 USA; 5 Department of Chemistry, Colorado State University, Fort Collins Colorado, 80523 USA; 6 University of Colorado Cancer Centre, Aurora Colorado, 80045 USA

## Abstract

Current antiretroviral therapy (ART) for HIV/AIDS slows disease progression by reducing viral loads and increasing CD4 counts. Yet ART is not curative due to the persistence of CD4+ T-cell proviral reservoirs that chronically resupply active virus. Elimination of these reservoirs through the administration of synergistic combinations of latency reversing agents (LRAs), such as histone deacetylase (HDAC) inhibitors and protein kinase C (PKC) modulators, provides a promising strategy to reduce if not eradicate the viral reservoir. Here, we demonstrate that largazole and its analogues are isoform-targeted histone deacetylase inhibitors and potent LRAs. Significantly, these isoform-targeted HDAC inhibitors synergize with PKC modulators, namely bryostatin-1 analogues (bryologs). Implementation of this unprecedented LRA combination induces HIV-1 reactivation to unparalleled levels and avoids global T-cell activation within resting CD4+ T-cells.

## Introduction

Viral latency in resting CD4+ T-cells remains the most important obstacle to reduction of the latent HIV reservoir in infected patients on anti-retroviral therapy (ART)^1–3^. This therapy reduces the active viral load in plasma to undetectable levels (<20 copies per mL). While effective for many, ART is costly and chronic, requires strict compliance, and is associated with early onset health problems arising from prolonged chemoexposure. Withdrawal of ART results in robust viral rebound from the T-cell reservoir of latent HIV-1 provirus, even in patients whose virus has remained undetectable for many years^4^. Elimination of the proviral reservoir if done in combination with ART would allow for eradication of HIV in ART-compliant individuals as well as a therapeutic strategy to address most HIV positive individuals who are non-compliant or do not have sustained access to ART.

One strategy for eradicating latent HIV is to activate proviral reservoir transcription with small molecule latency reversing agents (LRAs), while avoiding global T-cell activation which leads to cytokine release and toxicity^5, 6^. Upon activation of HIV transcription, the infected cells comprising the reservoir could be destroyed through viral cytopathic effects, host cytolytic mechanisms, immunotoxin or other therapeutic approaches^7^. Several compounds induce viral transcription and replication; however, some compounds, such as anti-T-cell receptor antibodies^8^, lead to global T-cell activation and are too toxic for use as latency reversing agents^9, 10^. Current efforts focus on LRAs that stimulate viral replication and avoid global T-cell activation^7, 11^.

Histone deacetylase (HDAC) inhibitors and protein kinase C (PKC) modulators represent two of the leading classes of small molecule LRAs. Several different HDAC inhibitors can reactivate HIV transcription and expression without global T-cell activation such as valproic acid (VPA), romidepsin and suberoylanilide hydroxamic acid (SAHA, vorinostat)^12–17^. Many PKC modulators have also been characterized as LRAs including ingenols^18^, prostratin^19–21^, 1,2 diacylglycerol analogs^22^, and bryostatin-1^23–25^. Bryostatin-1 has been used in phase I and phase II clinical trials as a therapeutic for many indications, including lymphoma, leukemia, Alzheimer’s disease, and most recently HIV^26–29^. For the cancer indications, bryostatin-1 is typically administered at 40–50 μg/m^2^ with myalgia being the dose-limiting side effect^26^. In the context of HIV latency reversal, bryostatin and other PKC modulators would be used at a lower minimum effective dose (MED) thereby allowing for a reduction in side effects. With regards to LRA potency, of the compounds studied, bryostatin-1 was the most effective in increasing HIV-1 mRNA levels close to those induced by strong T-cell activators in *ex-vivo* studies^25^.

**Figure 1:**
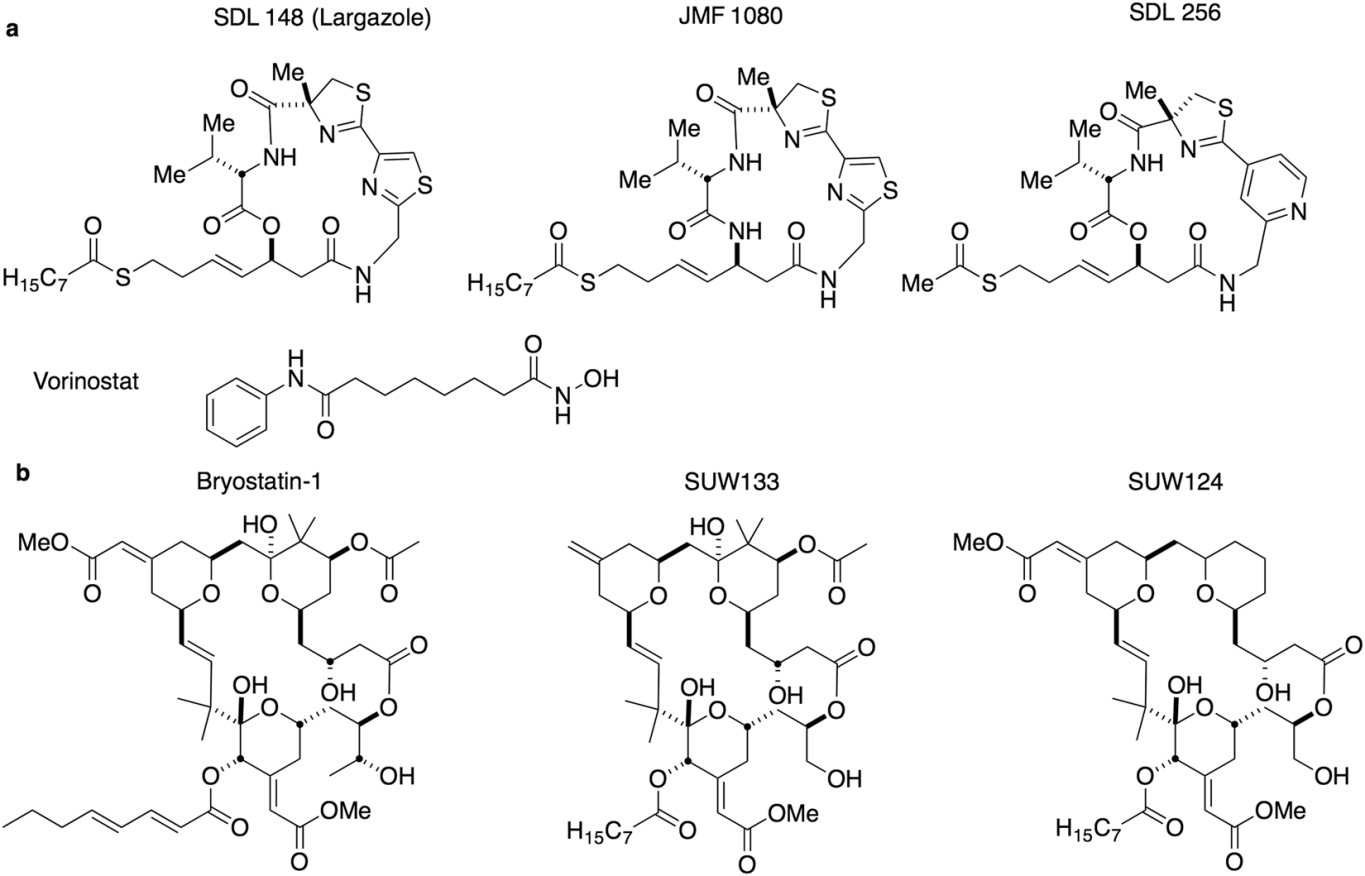
Structures of compounds implemented in LRA screens and HIV reactivation studies herein. (**a**) HDAC inhibitors including SDL148 (largazole), two of its derivatives JMF1080 and SDL256, as well as, vorinostat (SAHA). (**b**) PKC modulators including bryostatin-1 and two of its analogs SUW133 and SUW124.

LRA combination therapy involving both PKC modulators and HDAC inhibitors is more effective than separately using individual LRAs, both *in vitro* and in *ex vivo* blood draws^21, 23, 30^. Moreover, more effective combinations will lower the necessary dose of each component and would aid in the reduction of undesired side effects. Here, we demonstrate that largazoles are isoform-targeted class I HDAC inhibitors which efficiently reactivate HIV-1 from latently infected T-cells. Furthermore, given that newly designed and more synthetically accessible analogues of bryostatin-1 (bryologs) show better efficacy and tolerability as LRAs than the natural product itself *in vitro* and in animal models, we also show that largazoles display remarkable synergy when used in combination with bryologs (Fig. 1)^31–33^. This unprecedented LRA combination of bryologs together with largazole induces unparalleled levels of HIV expression and avoids global T-cell activation and cytokine release, making this combination a potentially strong therapeutic candidate for preclinical advancement.

## Results

### A screen of novel HDAC inhibitors identifies largazoles as low-toxicity HIV-1 latency reversing agents

A critical shortcoming of many leading HDAC inhibitors is an apparent lack of class specificity^34^. These pan-HDAC inhibitors, such as vorinostat, inhibit all classes of HDACs to similar degrees thus inviting the occurrence of untoward side effects. HDACs are divided into four classes (I-IV). The class I HDACs include HDAC1, -2, -3 and -8 while the class II HDACs incorporate HDAC4, -5, -6, -7, -9 and -10. Class III HDACs, known as sirtuins function through a different mechanism and have not been associated with HIV latency. The only member of class IV is HDAC11^35^. Accumulating evidence indicates HIV latency requires class I HDAC isoforms, especially HDAC1, -2 and -3, with HDAC3 being the most important^36, 37^. Therefore, isoform-targeted compounds for manipulation of a specific class I HDAC isoform could significantly figure in reducing untoward side effects^38–41^. However, it is unclear whether isoform-targeted compounds would prove as effective in reactivating HIV from latency as their pan-HDAC counterparts. To answer this question, we assayed several compounds, developed through virtual screening to be isoform-targeted HDAC inhibitors, for their ability to reactivate HIV from latency^42^. These HDAC inhibitors are comprised of a zinc binding group linked to a headgroup which interacts with amino acid residues at the edge of the inhibitor binding cavity of the HDAC^42^. Out of the 15 compounds screened (see Supplementary Table S1), largazole and two of its synthetic analogues emerged as potent LRAs. Initially isolated from marine cyanobacteria^43^, these cyclic depsipeptide compounds, known as largazoles, differentially affect cancer cell growth and demonstrate excellent bioavailability in mice^44–47^. Figure 1a shows the chemical structures of the three most potent largazoles used in this study.

**Figure 2:**
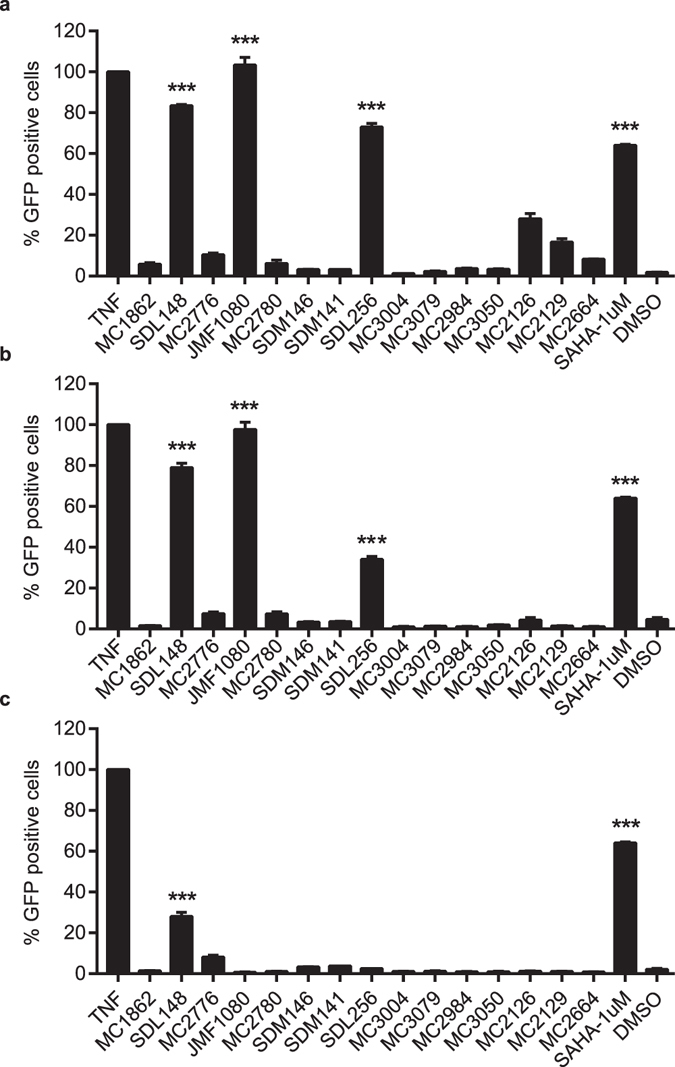
Screening of isoform-targeted compounds to identify HIV-1 latency reversing agents (LRAs). Identification of largazoles as potent LRAs. JLAT10.6 cells were incubated with 10 ng/mL of TNFα, 1 μM of SAHA (vorinostat) and 10 μM **(a)**, 1 μM **(b)**, or 0.1 μM **(c)** of screening compounds for 24 h. The percent of GFP positive cells were analyzed by FACS and the degree of HIV-1 reactivation was normalized to TNF-α. SDL148, JMF1080 and SDL256 are largazole and two analogs respectively. The mean values (mean ± s.e.m.) of a representative experiment in triplicate for each concentration is shown. ***p < 0.001 (ANOVA).

To screen the newly synthesized HDAC inhibitors, we employed JLAT10.6 cells, a T-cell line widely used for HIV-1 reactivation studies^48–50^. In this cell line, HIV-1Δenv, which arises from the NL4–3 backbone and contains a GFP reporter gene in place of the Nef gene, is stably integrated into the genome of Jurkat cells and expressed at undetectable levels at baseline. Upon reactivation of HIV transcription by stimulation with LRAs, GFP expression in proportion to viral production is determined by fluorescence activated cell sorting (FACS) analysis. Stronger HIV reactivation generates a more intense fluorescent signature per cell. In brief, the FACS analysis compares the number of cells with increased fluorescence to total number of cells scanned and reports this value as a percentage. We incubated JLAT 10.6 cells with HDAC inhibitor compounds for 24 hours at concentrations of 10, 1, and 0.1 μM. We used TNF-α and SAHA (vorinostat) as positive controls. At the given concentrations, SDL148 (largazole), and its derivatives JMF1080 and SDL256 induced the three highest activities for HIV-1 reactivation (Fig. 2). Although less potent than TNF-α, largazoles induce levels of HIV-1 activation comparable to, or better than that of SAHA, a currently accepted HIV-1 LRA. At 100 nM, SDL148 continued to significantly induce HIV-1 reactivation demonstrating superior potency compared to all other screened HDAC inhibitors.

**Figure 3:**
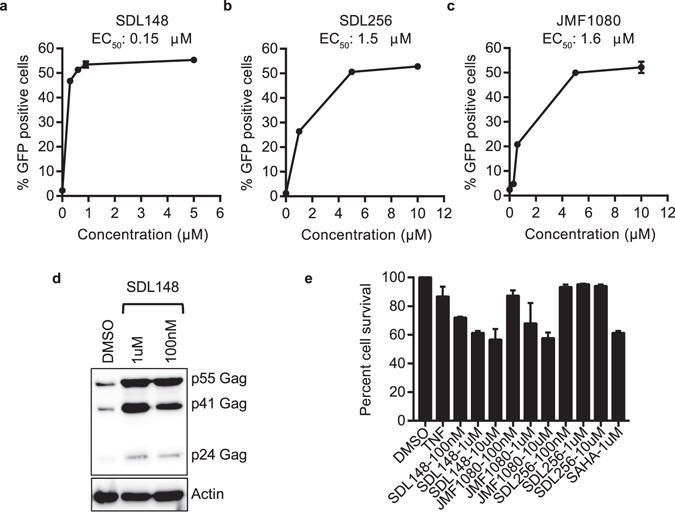
Low-toxicity isoform-targeted compounds induce HIV-1 gag. (**a–c**) EC_50_ measurements for largazoles. JLAT10.6 cells were incubated with indicated compounds for 24 h at increasing concentrations and FACS performed to quantify the percent of GFP positive cells. (**d**) Largazole induces HIV Gag proteins. JLAT10.6 cells were incubated with SDL148 for 24 h and Western blot performed for HIV Gag proteins. (**e**) Toxicity profile of largazoles. JLAT10.6 cells were incubated with compounds at indicated concentrations and MTT toxicity assay performed as described in Methods. Data indicates mean values, and error bars indicate mean ± s.e.m. (n ≥ 3).

We determined the EC_50_ for each of the three largazole compounds (Fig. 3a–c). With an EC_50_ of 0.15 μM, SDL148 exhibited the lowest EC_50_ among the three HDAC inhibitors followed by JMF1080 (1.6 μM) and SDL256 (1.5 μM). Consistent with its superior potency, SDL148 alone reactivates HIV expression significantly in the JLAT10.6 cells when used at 100 nM (Fig. 2c). Thus, we utilized SDL148 as an HDAC inhibitor LRA at 100 nM in the subsequent reactivation experiments involving LRA combinations in cell line models. We confirmed that the GFP expression seen with FACS analysis corresponds to enhanced expression of HIV Gag through Western blot assessment after incubation of JLAT10.6 cells with SDL148 for 24 h (Fig. 3d).

To determine the toxicity of these compounds on the JLAT10.6 cells, we used the MTT (3-(4,5-dimethylthiazol-2-yl)-2,5-diphenyltetrazolium) cell-proliferation assay^51^. *In-vivo*, only one in a million resting T cells have HIV proviruses, therefore resting T cells *in-vitro* without HIV infection is a good model for cellular proliferation analysis to assay toxicity. We found the toxicity of the three compounds to be similar to or less toxic than SAHA (Fig. 3e). These results show that the largazoles potently induce HIV-1 from latency with a toxicity profile comparable to that of the pan-HDAC inhibitor, SAHA.

### Largazoles reactivate HIV-1 in a primary cell model of latency

**Figure 4:**
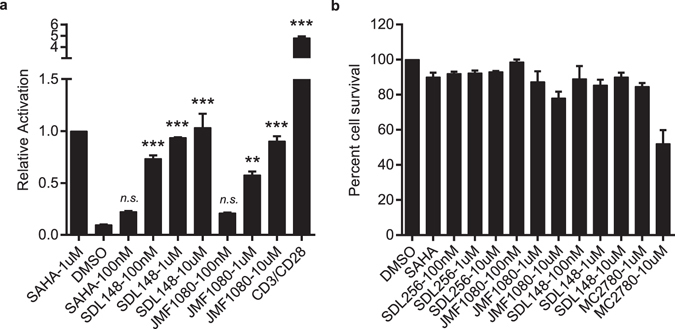
Largazoles induce HIV reactivation in primary cells. (**a**) A primary T-cell model of latency (See text for details). Latently infected T-cells were incubated with the indicated compounds for 48 h. HIV-1 reactivation is presented as fold induction relative to SAHA. Data indicate mean values of experiments in triplicate and error bars indicate mean ± s.e.m. (n ≥ 3). **p < 0.01, ***p < 0.001 (ANOVA). **(b)** Toxicity profile of largazoles in primary CD4+ T-cells. Primary resting CD4+ T-cells were incubated with compounds at indicated concentrations for 48 h and MTT toxicity assay performed as described in Methods. Percent cell survival was normalized to DMSO.

We then proceeded to test the ability of these compounds to reactivate HIV-1 in a primary cell model of latency. We used the primary cell model described by Greene and colleagues^52^ with a substitution of darunavir as a protease inhibitor, as described in the Methods section. Briefly, resting CD4 T-cells were isolated by negative selection, infected with an HIV-1 *Luc* reporter virus for 2 hours, washed and incubated for 72 h with the HIV-1 protease inhibitor darunavir. These cells produce no luciferase activity unless stimulated with appropriate agents. Cells were then stimulated for 48 h with the largazoles at indicated concentrations in the presence of the integrase inhibitor raltegravir, to prevent new integration. The level of reactivation was measured by luciferase luminescence read-out in cell lysates. Data for this model (Fig. 4a), show SDL148 and JMF1080 reactivate HIV-1 in a dose-dependent manner indicating that largazoles are potent LRAs. Again, the most potent compound was SDL148 which performed similar to SAHA at a 10-fold lower concentration. After 48 h of incubation, the toxicity levels of the largazoles were assessed in resting CD4 T-cells. Compared to an inactive compound, MC 2780, the toxicity levels induced by largazoles were minimal as determined by MTT cell-proliferation assay (Fig. 4b).

### Largazoles are class-targeted HDAC inhibitors that remodel chromatin at the HIV-1 promoter

**Figure 5:**
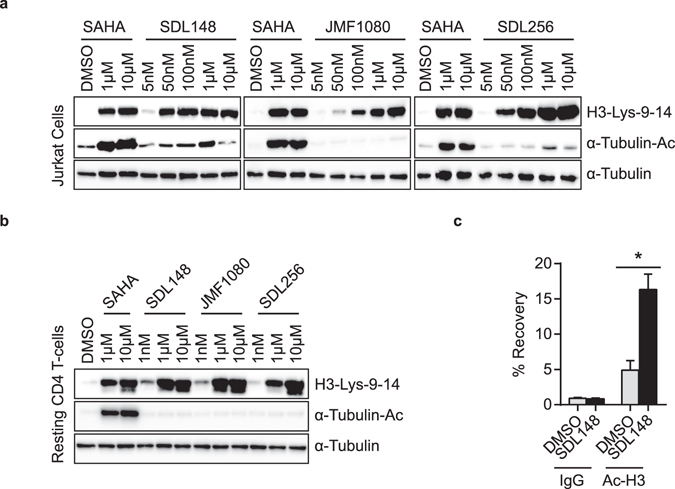
Largazoles are isoform-targeted class I HDAC inhibitors that enhance histone H3 acetylation at the HIV promoter. Largazoles inhibit class I, but not class II histone deacetylases. Compounds at indicated concentrations were incubated with **(a)** Jurkat or **(b)** primary T-cells for 8 h and Western blot performed for acetylated histone H3 (a marker of class I HDAC inhibition) or acetylated tubulin (a marker for HDAC6, a class II HDAC) inhibition. **(c)** Chromatin immunoprecipitation (ChIP) of acetylated histone H3 and HIV LTR promoter. JLAT10.6 cells were incubated with DMSO or 1 μM of SDL148 for 6 h and ChIP performed as described in Methods. Data indicate mean values and error bars indicate mean ± s.e.m. (n ≥ 3). *p < 0.05, (Student’s *t*-test). See also Supplementary Fig. S1.

We determined the class specificity of the three potent largazole analogues (Fig. 1) through Western blots on Jurkat cell lines and primary resting CD4+ T-cells following 8 hours of incubation with increasing concentrations of the HDAC inhibitors. We probed the Western blots for acetylated histone H3, a measure of class I HDAC inhibition, and acetylated tubulin, a measure of HDAC6 (class II HDAC) inhibition^47, 53^. The largazole compounds specifically enhanced histone H3 acetylation while SAHA, a pan-HDAC inhibitor enhanced the acetylation of both histone H3 and tubulin (Fig. 5a,b). At 50 nM, SDL148 yields levels of acetylated histone H3 which are similar to those induced by SAHA at 1 μM. This observation further demonstrates the superior potency of SDL148, now in a histone acetylation assay, and is consistent with previous data (Fig. 2). The class I HDAC inhibitory activity demonstrated by the largazoles is not restricted to T-cells, as similar inhibitory activity is seen in HeLa cells (Supplementary Fig. S1). In primary T-cells, the largazoles did not induce detectable acetylated tubulin levels when employing concentrations up to 10 µM; however, much lower concentrations of SAHA lead to acetylation of tubulin (Fig. 5b). This data confirms the largazoles as class 1 targeted HDAC inhibitors in T-cells.

Since histone H3 is deacetylated by class I HDACs and known to bind to the HIV LTR promoter, we determined H3 acetylation status of the HIV-1 LTR in JLAT10.6 cells. We incubated a DMSO control and a SDL148 test with JLAT10.6 cells for 6 hours and performed chromatin immunoprecipitation (ChIP) assays. Using ChIP, we recovered 15% of the DNA from SDL148-treated cells using anti-acetylated histone H3 antibody compared to less than 5% recovery with DMSO-treated cells, and less than 1% from the IgG-negative controls (Fig. 5c). This shows that largazole actively remodels the chromatin at the HIV-1 LTR which could explain the reactivation of the virus. An alternative mechanism is modulation of lysine residues in Tat which impact its ability to interact with TAR of the HIV-1 LTR^54–56^. Taken together, these findings confirm largazoles as HDAC inhibitors that preferentially target class I histone deacetylases with the ability to activate the HIV-1 LTR to overcome latency.

### Largazole combinations with bryologs show synergy and unprecedented potency as HIV latency reversing agents

Bryostatin-1 has been shown to display synergistic effects with vorinostat and other HDAC inhibitors^30^. However, bryologs have proven to be more potent activators of latent HIV reservoirs^32^, and we have now characterized SDL148 as a more potent LRA than vorinostat. Therefore, to produce a treatment which most effectively reverses HIV latency, we evaluated a combination of the leading LRAs (Fig. 1b) of both PKC modulators (SUW133, SUW124) and HDAC inhibitor (SDL148).

**Figure 6:**
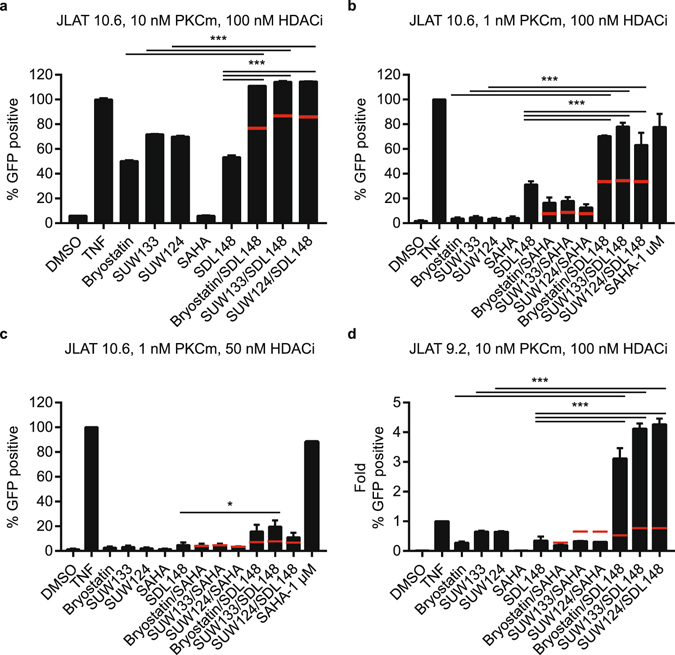
Largazole combinations with bryologs show synergy and unprecedented potency as HIV latency reversing agents. JLAT cell lines were incubated with different concentration combinations of LRAs. The percent of GFP positive cells was analyzed by FACS and the degree of HIV-1 reactivation was normalized to TNF-α. 10 ng/mL TNF-α was used as the positive control. JLAT 10.6 cells (**a**–**c**) and JLAT 9.2 cells (**d**) were incubated for 18–24 h with (**a**) 10 nM PKC modulator (PKCm) and 100 nM HDAC inhibitor (HDACI) concentrations, (**b**) 1 nM PKCm and 100 nM HDACI, (**c**) 1 nM PKCm and 50 nM HDACI, (**d**) 10 nM PKCm and 100 nM HDACI. Panel (**d**) presented as a fold change relative to TNF-a. Data indicate mean values of experiments in duplicate (Fig. 4a) or triplicate (Fig. 4b–d) and error bars represent mean ± s.e.m. (n ≥ 3). *p < 0.05, ***p < 0.001 (ANOVA). Red-coloured dashes indicate the predicted fractional response based on the Bliss independence model.

In these combination LRA studies, shown in Fig. 6, we utilized the aforementioned Jurkat T-cell lines, JLAT 10.6 and JLAT 9.2, to probe the ability of the novel LRA combination to reverse HIV-1 latency. In Fig. 6, we see the observed fractional response of the LRA combinations, as indicated by the black bars, compared to the predicted fractional response as predicted by the Bliss independence model and indicated by the red-coloured dashes for each LRA combination (see Methods for details and Supplementary Fig. S2)^57^. We first incubated JLAT 10.6 cells for 18–24 hours with various concentrations of LRAs and used TNF-α as the positive control. In line with their greater efficacy, bryologs display stronger activation compared to bryostatin-1 when incubated with JLAT 10.6 cells at 10 nM (Fig. 6a). Combination with 100 nM HDAC inhibitor, SDL148, induced significantly higher levels of activation than bryologs or largazole alone. At these concentrations, the LRA combinations induce activation levels equal to or better than that induced by TNF-α.

Next, we reduced the PKC modulator concentration to 1 nM, and maintained the HDAC inhibitor concentration at 100 nM. With reduced PKC modulator concentration, we observed large amounts of reactivation due to synergistic effects from the combination of bryologs with largazole (Fig. 6b). The synergy of this novel combination at these concentrations leads to significant activation levels comparable to TNF-α. Combinations of the bryologs and the pan-HDAC inhibitor, SAHA, also displays synergy, but to a much lesser degree. Reduction of the HDAC inhibitor concentration to 50 nM greatly reduces the overall number of cells expressing appreciable levels of GFP; however, the combination of bryologs and largazole still leads to synergistic effects (Fig. 6c), and the SUW133/SDL148 combination still exhibits a significant level of reactivation compared to SDL148 alone.

The environment at the integration site affects the HIV-1 transcriptional rate^58, 59^. Reactivation assays employing a separate Jurkat T-cell line (JLAT 9.2 clone), with a proposed different integration site based on viral transcriptional rates, allowed us to explore the effect of heterogeneous HIV integration and gain a better understanding of the potency of our novel LRA combination^50^. We incubated JLAT 9.2 cells with 10 nM PKC modulator and 100 nM HDAC inhibitor for 24 hours (Fig. 6d). We utilized TNF-α as a positive control and assayed viral activation by FACS. These combinations of bryologs with largazole induce HIV reactivation four times more strongly than TNF-α. The remarkable amount of synergy, and overall activation of the latent virus, demonstrates the superiority of this combination. To the best of our knowledge, the combination of bryologs (SUW133 and SUW124) with largazole is currently the most potent LRA combination for HIV-1 latency reversal in the JLAT cell lines.

### Synergistic combinations avoid global T-cell activation and induction of cytokine release

**Figure 7:**
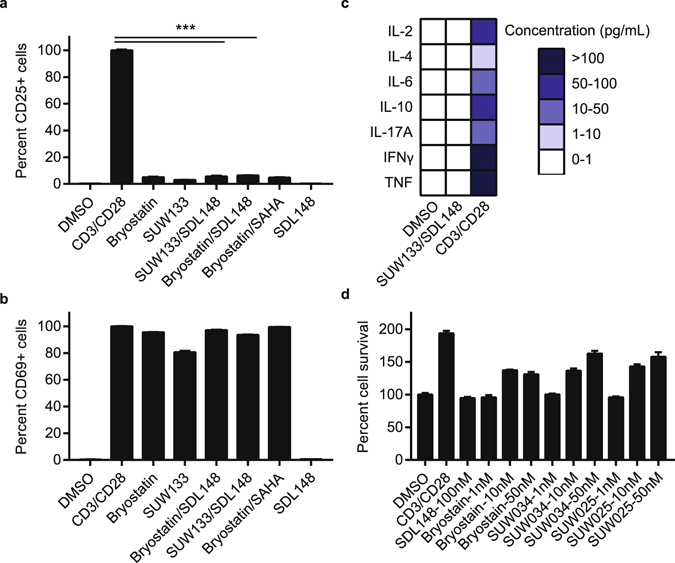
Synergistic combinations avoid global T-cell activation and induction of cytokine release. Resting CD4+ CD25− T-cells were isolated from a HIV-1 negative donor blood. Cells were incubated 24 h with 1 nM bryologs and 100 nM HDAC inhibitor. Cells were stained with fluorescent-conjugated antibodies **(a)** CD25-APC and **(b)** CD69-FITC and analyzed by FACS (see Methods). Data indicate mean values and error bars indicate mean ± s.e.m. (n ≥ 3). ***p < 0.001, (ANOVA). **(c)** ELISA results for cytokine release from CD4+ CD25- T-cells following 24 h incubation with LRA combinations (see Methods). (**d**) Toxicity profile of LRA combinations. Resting CD4+ T-cells were incubated with compounds at indicated concentrations and MTT toxicity assay was performed as described in Methods.

To investigate global T-cell activation and potential cytokine release, we used primary CD4 + CD25(−) T-cells isolated from HIV negative donor blood (see Methods). We probed the CD4 + CD25(−) T-cells for cell surface expression of CD25, a late T-cell activation marker, and CD69, a very early stage activation marker after stimulation with the most potent LRA combination, SUW133 with SDL148. Individual compounds and the novel LRA combinations induce little to no expression of CD25 (Fig. 7a) and expression of CD25 following stimulation by the positive control, CD3/CD28, is significantly increased compared to the LRA combinations. Expression of increased levels of CD69 following stimulation with PKC modulators or LRA combinations demonstrate some amount of very early T-cell activation (Fig. 7b), but surprisingly stimulation with SDL148 alone does not lead to any significant CD69 expression.

In Fig. 7c, we used ELISA to probe the supernatant from resting T-cells for cytokine release (see Methods). In brief, CD4 + CD25(−) resting T-cells were stimulated with the most potent LRA combination as determined by FACS analysis (Fig. 6). The supernatant from these cells following 24 h stimulation by SUW133 with SDL148 was collected and probed for a range of different cytokines as indicated in Fig. 7c. Although, high concentrations of cytokines were observed when resting T-cells were stimulated with CD3/CD28, the SUW133 with SDL148 combination induced no detectable levels of cytokines. The limited expression of CD25, the absence of any cytokine release, and no perceived cell death seen in the MTT cell proliferation assays (Fig. 7d) suggest that stimulation by these LRA combinations at indicated concentrations avoids sustained T-cell activation associated with global activation, cytokine recruitment, and overall toxicity.

## Discussion

The development of isoform-targeted LRAs will aid in the understanding of molecular mechanisms which reverse HIV latency and lead to fewer untoward side effects. Most of the current leading HDAC inhibitors affect many, if not all, of the eight zinc-based HDAC isoforms making it difficult to ascertain which HDACs are responsible for promoting HIV-1 latency. While protein-knockdown assays have been essential in narrowing down the HDACs that are important for HIV latency, these assays can only be done in cell lines amenable to effective transfections or transductions. Identification of isoform-specific HDAC inhibitors will help to decipher the contributions of a particular HDAC in an individual patient. Although the assays performed herein do not isolate one of the specific class I HDACs as responsible for promoting HIV-1 latency, previous work has indicated preference of largazole to inhibit HDAC1, followed by HDAC3 and HDAC2^45^. It is quite feasible that inhibition of more than one HDAC is required for optimal activation; therefore, further development and investigation of HDAC inhibitors would be advantageous.

We conclude that largazoles are isoform-targeted due to their ability to inhibit class I HDACs (stimulate histone H3 acetylation) at concentrations of 50 nM, while doses up to 10 µM had no detectable effect on class II (HDAC6) activity in primary T-cells. The fact that largazole performed similarly or better than vorinostat in reactivation assays confirms that isoform-targeted compounds could be more effective than their pan-HDAC inhibitor counterparts. Largazoles have been tested in mice and found to have a very low toxicity profile^47^. While they are yet to be tested in humans, it is conceivable that they will have very few side effects based on inhibition of fewer histone deacetylases.

Although it is not definitive whether largazole actively remodels the chromatin at the HIV-1 LTR or modulates the Tat lysines to limit Tat/TAR interactions, it does synergize extremely well with bryologs, resulting in four times higher reactivation of HIV in the JLAT 9.2 cell line compared to the positive control, TNF-α. Given these achievements of the novel LRA combination in HIV-1 T-cell line models, we sought to explore their effects within primary CD4+ T-cells extracted from HIV negative donors. CD69 is upregulated when the NF-kB pathway is activated in a PKC-dependent manner^60^. Knowing that the PKC modulators herein bind PKC, it is reasonable to see that after the course of 24 hours of activation, CD69 cell surface markers are increased. However, the late stage T-cell activation marker, CD25, is largely absent at 24 hours. Furthermore, we observe no detectable cytokine release or notable toxicity.

In summary, we have shown that an unprecedented, potent LRA combination greatly enhances the activation of latent HIV-1 proviral reservoirs as is required for their elimination. The largazoles used in this study are class I targeted HDAC inhibitors that initiate increased HIV-1 expression in comparison to their pan-HDAC inhibitor counterparts. The bryologs used in this study are more effective activators than the natural product bryostatin-1. The first tested combination of these two LRA classes (largazoles and bryologs) show great promise for latency reversal as they operate with impressive synergy and exhibit pronounced efficacy for reactivation of latent HIV. These novel combinations avoid global T-cell activation, induce no detectable levels of cytokine release, and show no notable toxicity in CD4+ T-cells. These findings bode well for their use *in vivo*, and provides a foundation for the projected advancement of this combination toward clinical trials.

## Methods

### Primary cell studies

The Washington University Institutional Review Board approved this study, and all patient participants who donated blood samples for primary cell isolation gave written informed consent for this study. All methods and experiments were performed in accordance to the guidelines and regulations outlined by the Washington University Institutional Review Board.

### Cell lines

JLAT 10.6 and JLAT 9.2 cells were obtained from the NIH AIDS Reagent Program, Division of AIDS, NIAID. The JLAT clones originally developed by Jordan and colleagues^50^. Briefly, lymphocytic cell line Jurkat was infected with HIV-R7/E^−^/GFP and were then sorted with differential FACS based on GFP expression. These cells demonstrate viral transcriptional silence at baseline. JLAT and Jurkat cells were maintained in RPMI-1640 supplemented with heat inactivated fetal bovine serum; L-glutamine, 200 nM; penicillin/streptomycin, 100x solution; and sodium pyruvate (10%, 2%, 1%, and 0.5% by volume).

### Cell line screen of isoform-targeted histone deacetylase inhibitor compounds

JLAT10.6 cells were treated with HDAC inhibitor compounds for 24 h. The concentrations of the compounds are shown in the respective figure legends. After a dose response determination, we found 1 μM of vorinostat (SAHA) to be the most effective concentration to use in JLAT10.6 and primary cell models. JLAT10.6 cells were treated for 24 h and GFP positive cells were observed by FACS analysis using the Becton Dickinson (BD) FACS machine. Data was analysed with BD’s CellQuest Pro software. The cells typically diverged into two groups on the scatterplot based on forward scatter and side scatter light intensity. Healthier cells to the right of the plot were selected across treatments for analysis. Drug candidates were identified as positive if the amount of GFP expression induced was equal to or greater than 10% of the reactivation induced by TNF-α.

### Cellular Toxicity Assays

To test the toxicity produced by the positive drug candidates for the HDAC inhibitor screen, a 3-(4, 5-dimethylthiazolyl-2)-2, 5-diphenyltetrazolium bromide (MTT) assay was used. An MTT assay is a colorimetric assay that can be used to assess cell viability. The assay relies on the measurement of the presence of NAD(P)H-dependent cellular oxidoreductase enzymes, which are present in cellular mitochondria. The presence of these enzymes and thus the presence of active mitochondria is a reliable indicator of cellular viability. These enzymes reduce the MTT dye to formazan, which has a purple colour, allowing the amount of these enzymes, and by extension the number of viable cells, to be quantified. The JLAT 10.6 Cells were exposed to the same experimental conditions described above, but were re-suspended in MTT dye (Sigma Aldrich) instead of paraformaldehyde. The MTT dye stains mitochondria. After 3–4 hours of incubation with the MTT dye, the cells were lysed using the solubilisation buffer. The absorbance of each sample was reported by a microplate reader (TECAN), using the Magellan software program at a wavelength of 570 nm with a reference wavelength of 750 nm.

The MTT assay was performed in the same manner to assess the toxicity of the synergistic LRA combinations of the bryologs with largazole at the concentrations indicated in the figure legends.

### Western Blots

Jurkat, HeLa or primary CD4+ T-cells were incubated with compounds for 8 hours washed in PBS and lysed with buffer containing 0.2% NP40 and protease inhibitor cocktail (Roche). Twenty micrograms of protein were loaded and separated on a 12.5% SDS-polyacrylamide gel and transferred to nitrocellulose. The membrane was probed overnight at 4 °C in 5% milk in PBS/Tween 20 (0.05%). After washing with PBS/Tween, the blot was probed with appropriate horseradish peroxidase-conjugated secondary antibody for 1 hour at room temperature and stained with Femto Supersignal. Tubulin or actin was used as loading control as indicated.

### Primary cell latency model

We used the Greene primary cell latency model, but substituted darunavir as the protease inhibitor^52^. Briefly, isolated CD4+ T cells (see primary cell isolation and culture) were infected with replication competent HIV-1 NL4–3 Luciferase virus (kind gift from Warner Greene) under spinoculation. Cells were then washed, incubated with 10 μM of the protease inhibitor darunavir (Selleckchem) for 48 h. Darunavir is a more potent protease inhibitor and does not affect the cell latency model. Cell was washed again and a million cells incubated with various concentrations of compounds for 48 h in the presence of the integrase inhibitor raltegravir to prevent new integration events. Cells were then lysed and luciferase luminescence determined as a measure of viral reactivation from latency.

### Chromatin Immunoprecipitation (ChIP) Assay

JLAT10.6 cells were treated with DMSO or 1 µM of SDL148 for 6 h. ChIP was performed with a ChIP kit from Abcam (ab500) and the anti-acetylated histone H3 antibody (Thermo Scientific). An anti–rabbit IgG (Cell Signaling Technology) was used as an isotype control. Total chromatin (input) and the immunoprecipitated chromatin were used as template, and the HIV-1 LTR was amplified by quantitative PCR using the primers indicated. Actin was used as internal control. We used the HIV-1 LTR primers (f: 5-AGCCCTCAGATG CTACATATAAGCA-3, r: 5-TAG CCAGAGAGCTCCCAGGCTCAG A - 3) and actin primers.

### Cell line screen of protein kinase C modulator compounds and flow cytometry of synergistic combinations

JLAT10.6 or JLAT 9.2 cells were treated for 18–24 hours and GFP positive cells were observed by FACS analysis (see cell line screen of isoform-targeted histone deacetylase inhibitor compounds Methods section). The concentrations of the PKC modulator compounds are shown in the respective figure legends.

### Primary cell isolation and culture

Peripheral blood mononuclear cells were isolated from blood of HIV negative donors using density gradient centrifugation through a Ficoll-Hypaque gradient (GE Healthcare). The EasySep CD4+ T-cell Enrichment Kit (STEMCELL Technologies) was used to isolate CD4+ T-cells. Resting CD4+ T lymphocytes were further enriched by depletion of cells expressing CD25 by negative selection (STEMCELL Technologies). We verified the purity of resting CD4 + lymphocytes by flow cytometry and typically found it to be greater than 98%. Isolated cells were maintained in RPMI-1640 supplemented with 10% FBS.

### Expression of activation markers in primary resting CD4+ T-cells

CD4+ T-cells were acquired (see Primary cell isolation and culture Methods section) from HIV negative donor blood. Surface expression of CD25 and CD69 activation makers was analysed by FACS. These cells were cultured for 24 hours with the following drug conditions in triplicate −12.5 µL anit-CD3/CD28 beads, 50 ng/5 µg per mL of PMA/Ionomycin, 1 nM bryologs and 100 nM of largazole. Each replicate contained one million CD4+ T-cells. Post 24-hour activation, fluorescently-conjugated antibodies targeting CD25, and CD69 were used to stain replicates. Antibodies were incubated with cells at 4 °C for 20 minutes then washed with PBS before running FACS analysis. The same BD FACS machine was used as mentioned above. Similar analysis was done based on fluorescence of the respective tethered fluorophore.

### ELISA

CD4 + CD25− T-cells (see primary cell isolation and culture Methods section) were incubated with various concentrations of compounds for 24 h. Cells were then spun down and supernatants were collected and stored at −20 °C. We performed ELISA per the enclosed protocol inside the Multi-Analyte ELISArray Kit (MEH-004A, QIAGEN) to detect cytokine release from activated T-cells. The 96-well plate used in the ELISA was read at 450 nm for absorbance and 570 nm for reference.

### Chemicals

Tumour necrosis factor alpha (TNF-α), bryostatin-1, vorinostat, phorbol 12-myristate 13-acetate (PMA), Ionomycin were obtained from Sigma. Dynabeads Human T-Activator CD3/CD28 (CD3/CD28) were purchased from ThermoFisher Scientific.

Fluorescently-conjugated antibodies for the flow cytometry, FITC-conjugated anti-human CD69 (clone FN50, Catalogue No. 11-0699-41) and APC-conjugated anit-human CD25 (clone BC96, Catalogue No. 17-0259-41), were obtained from eBiosciences. The isolation and synthesis of largazole has previously been described^43, 61, 62^. The HDAC inhibitors were designed with a computational ligand screening analysis based on comparative binding energy^39^. The organic synthesis for the largazole analogues^42^ and bryologs^32, 33^ is described previously.

### Bliss Independence

We use the Bliss independence model, one method to predict combined effects from multiple drug applications with the assumption that the drugs behave through independent mechanisms, to analyse the latency reversing agent combinations. Bliss independence is defined by *f*_ab,P_ = *f*_a_ + *f*_b_ − (*f*_a_ *f*_b_)^30, 57^. The predicted fractional response (*f*_ab,P_) is found per the Bliss independence model by taking the observed fractional response of drug A (*f*_a_), adding it to the observed fractional response of drug B (*f*_b_) and subtracting the fractional response that might arise from a simultaneous response of drug A and drug B (*f*_ab_). (*f*_ab_). The fractional difference (Δ*f*) is then calculated by taking the difference between the observed fractional response (*f*_ab,O_) and the predicted fractional response (*f*_ab,P_). Positive results indicate synergistic effects, negative results indicate antagonistic effects, and equality indicates true independence.

### Statistics

The mean values of data were compared using either Student’s t-test for pairwise comparisons or a one-way analysis of variance (ANOVA) with a follow-up Turkey multiple comparisons test for multiple comparisons. The *p*-values < 0.05 were considered significant. Calculations were performed with GraphPad PRISM 7.00 (GraphPad Software, Inc, La Jolla, CA).

## Electronic supplementary material

Supplementary Information
