# Supplementary material for: Combinations of isoform-targeted histone deacetylase inhibitors and bryostatin analogues display remarkable potency to activate latent HIV without global T-cell activation
Source: Sci Rep. 2017 Aug 7;7:7456. doi: 10.1038/s41598-017-07814-4 (PMC5547048; doi:10.1038/s41598-017-07814-4)
Supplement: Supplementary file 1 — Supplementary Information [file 41598_2017_7814_MOESM1_ESM.pdf]

Supplementary Information

Combinations of isoform-targeted histone deacetylase inhibitors and bryostatin analogues display remarkable potency to activate latent HIV without global T-cell activation.

Brice J. Albert, Austin Niu, Rashmi Ramani, Garland R. Marshall, Paul A. Wender, Robert M. Williams, Lee Ratner, Alexander B. Barnes, George B. Kyei

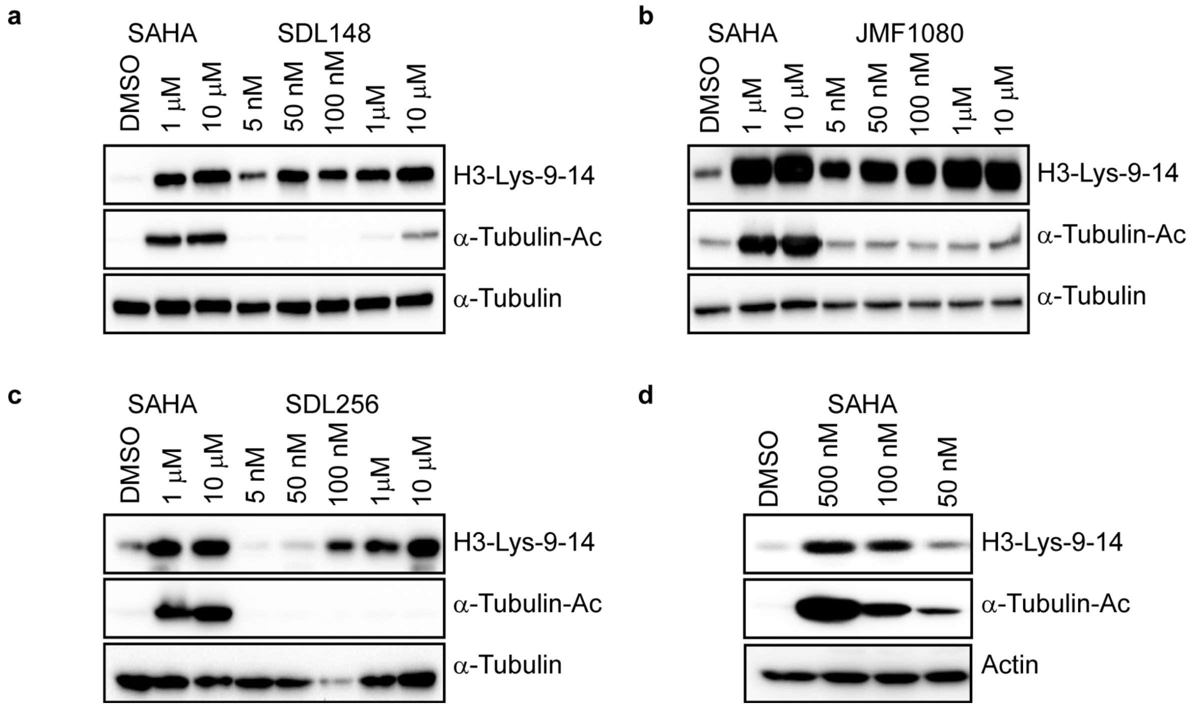

Supplementary Figure S1. HDAC inhibition in HeLa and primary T-cells.
Compounds at indicated concentrations were incubated with HeLa cells **(a-c)** or primary T-cells **(d)** for 8h and WB performed for acetylated histone H3 (a marker of class I HDAC inhibition) or acetylated tubulin (a marker for HDAC6, class II HDAC) inhibition. Related to Fig. 5.

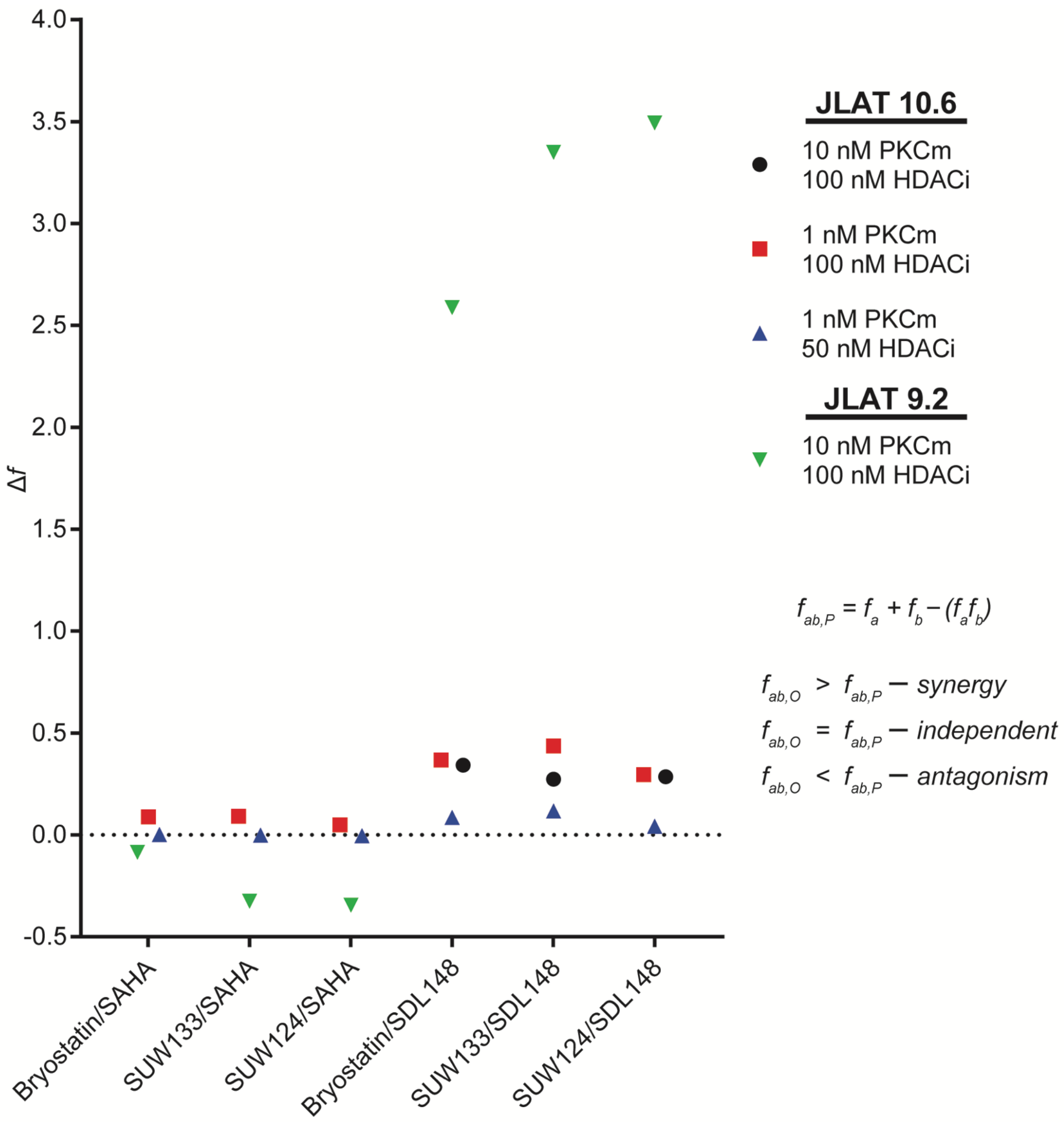

Supplementary Figure S2. Combination LRA fractional differences per Bliss independence prediction. The fractional difference (Δ*f*) is calculated by taking the difference between the observed fractional response ($f_{ab,O}$) and the predicted fractional response ($f_{ab,P}$) to determine if the two drugs are truly independent. The predicted fractional response ($f_{ab,P}$) is found per the Bliss independence model by taking the observed fractional response of drug A (f_a), adding it to the observed fractional response of drug B (f_b) and subtracting the fractional response that might arise from a simultaneous response of drug A

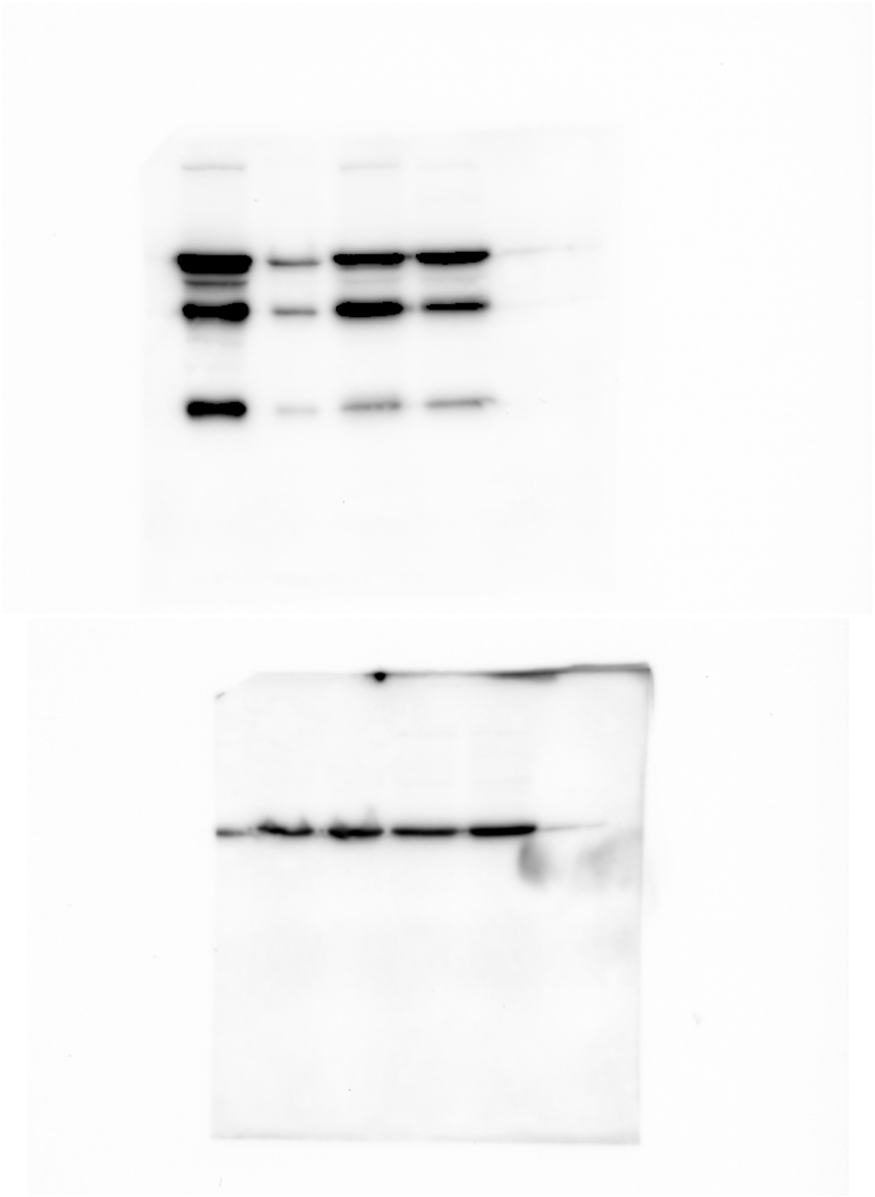

Supplementary Figure S3. Full unedited gels shown in Figure 3D of manuscript. (Top) The right three lanes are shown in the figure. (Bottom) Actin blot for the same gel.

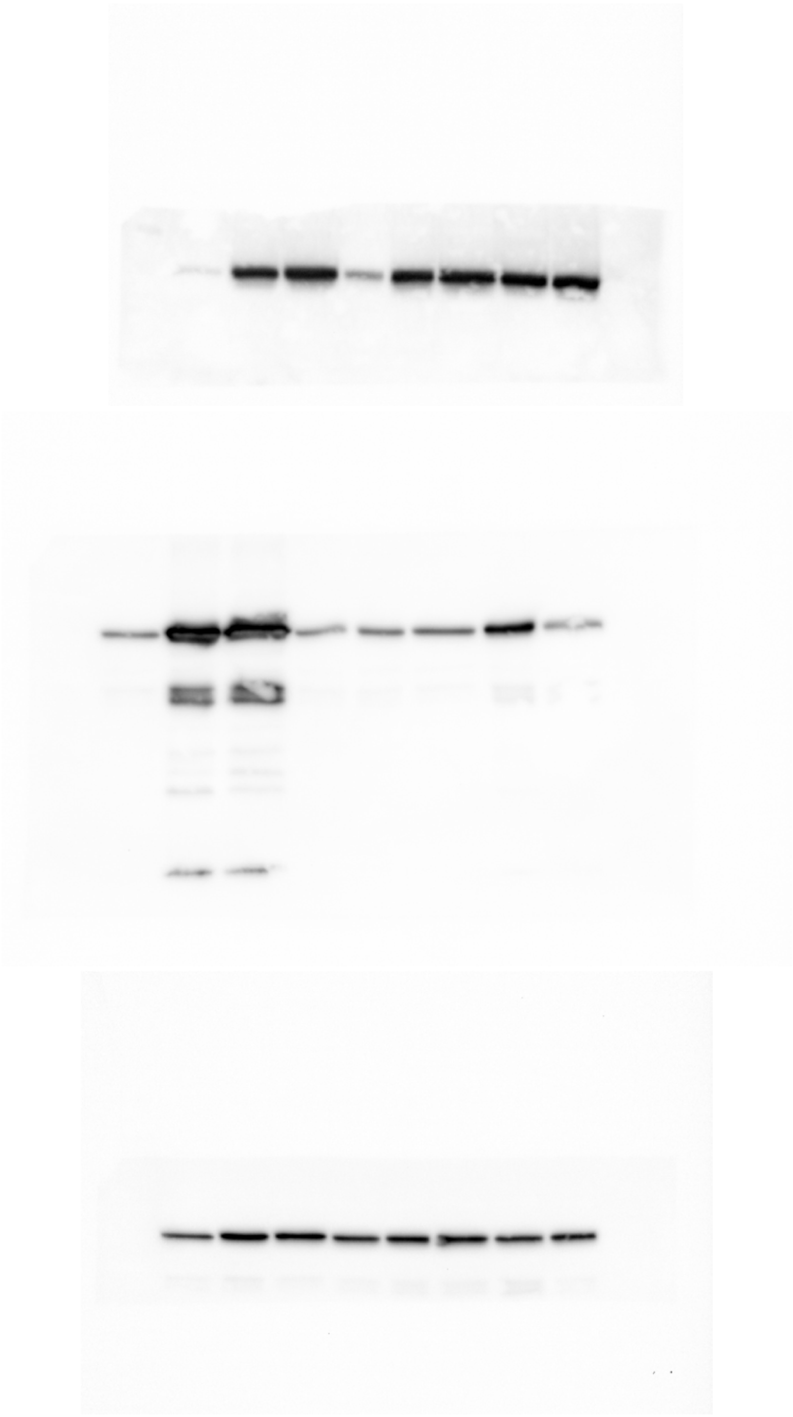

Supplementary Figure S4. Full unedited gels shown in Figure 5a, left (SDL148) of manuscript. (top) H3-Lys-9-14. (center) α-Tubulin-Ac. (bottom) α-Tubulin

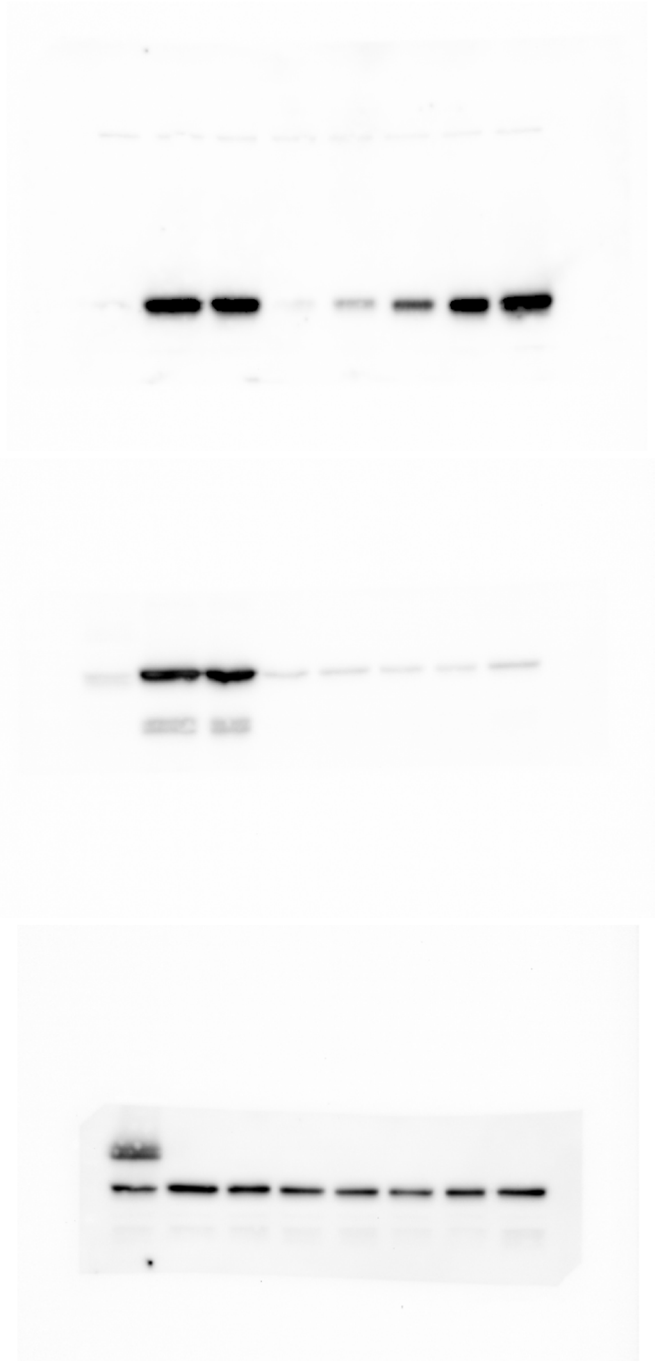

Supplementary Figure S5. Full unedited gels shown in Figure 5a, center (JMF1080) of manuscript. (top) H3-Lys-9-14. (center) α-Tubulin-Ac. (bottom) α-Tubulin

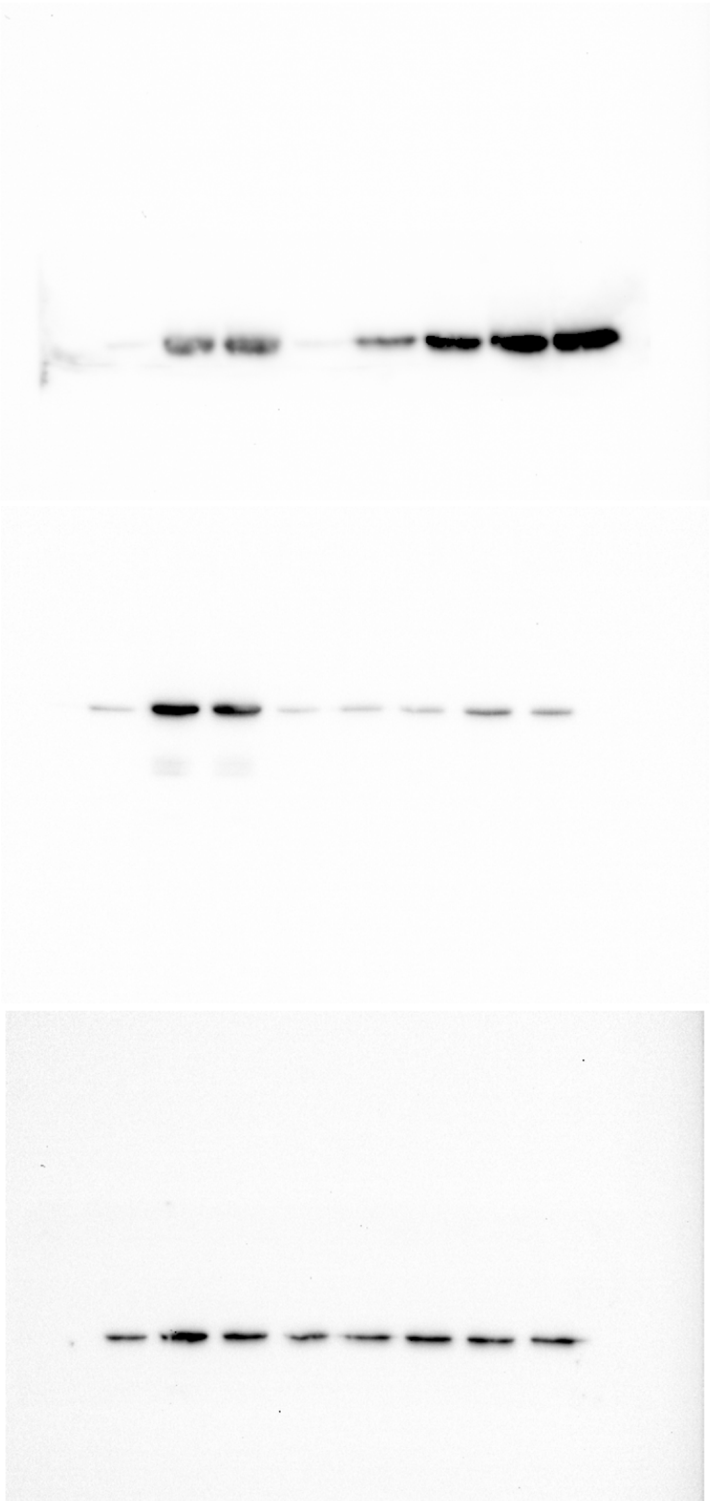

Supplementary Figure S6. Full unedited gels shown in Figure 5a, right (SDL256) of manuscript. (top) H3-Lys-9-14. (center) α-Tubulin-Ac. (bottom) α-Tubulin

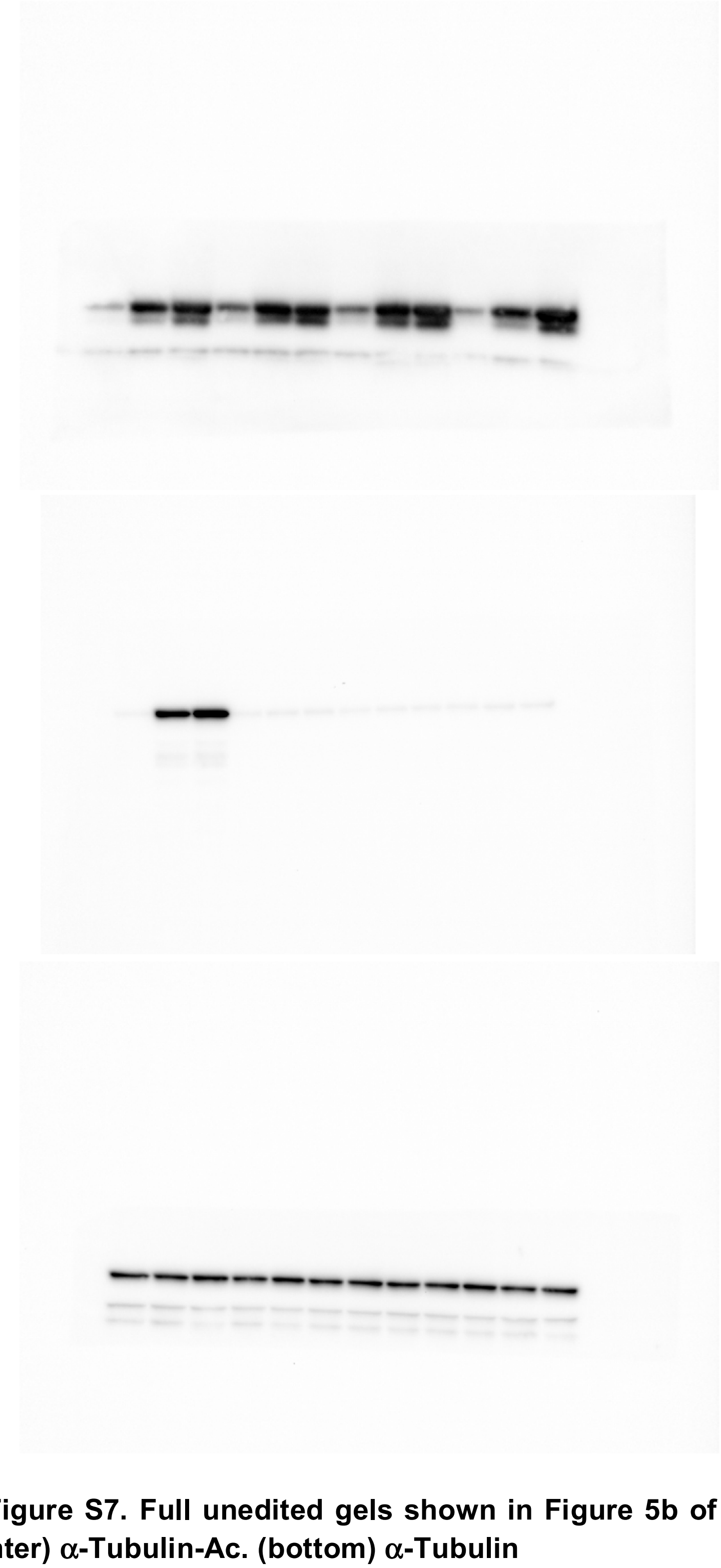

Supplementary Figure S7. Full unedited gels shown in Figure 5b of manuscript. (top) H3-Lys-9-14. (center) α-Tubulin-Ac. (bottom) α-Tubulin

Supplementary Table 1. Compounds developed through virtual screening to be isoform-targeted HDAC inhibitors. Related to Figure 2.

Compounds	Molecular Structure	Molecular Weight	clogP
**MC2984**	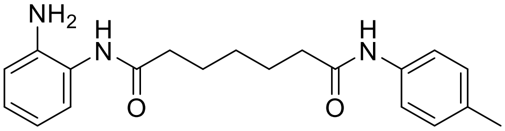	339.43	2.49
**SDM141**	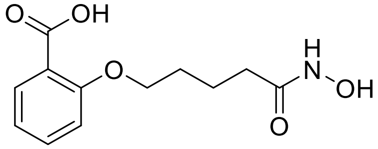	253.25	0.36
**SDM146**	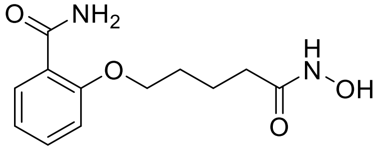	252.27	-0.46
**MC2727**	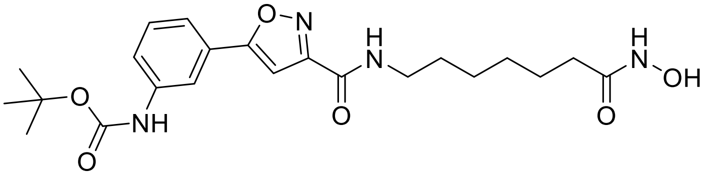	446.50	2.41
**MC2726**	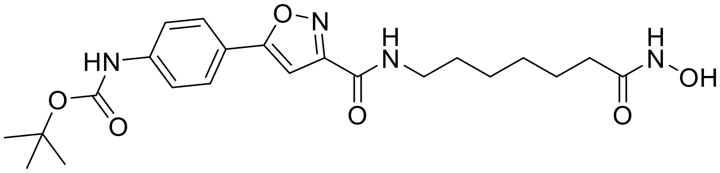	446.50	2.41
**MC2625**	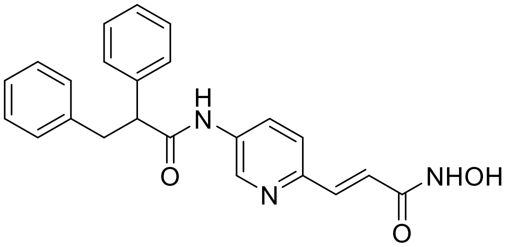	387.43	2.50
**MC2664**	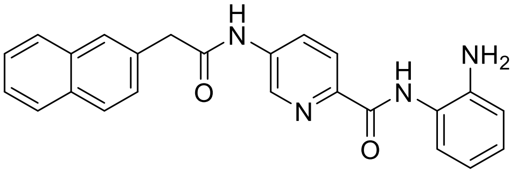	396.44	3.61
**MC2780**	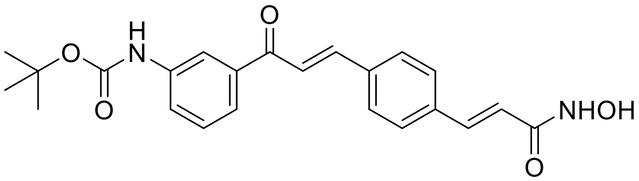	408.45	3.16

**MC2776**	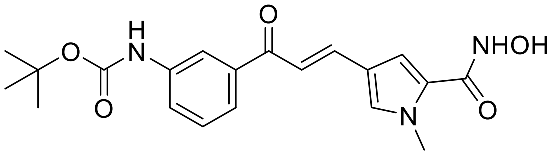	385.42	2.52
**MC3031**	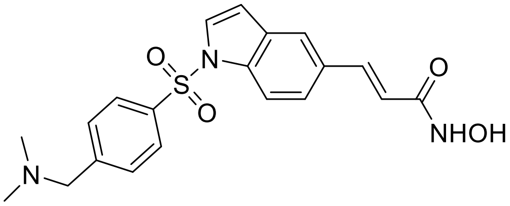	399.46	2.06
**MC3004**	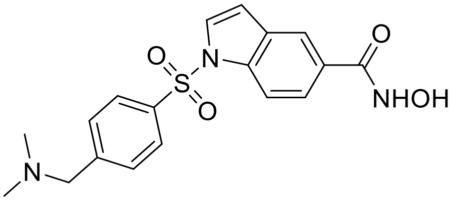	373.43	2.07
**MC3079**	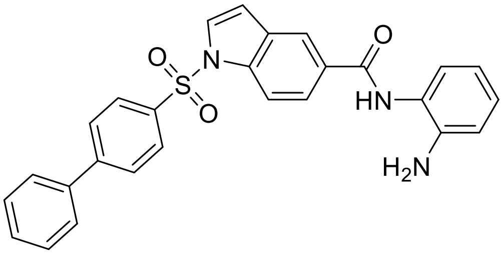	467.54	5.29
**MC3050**	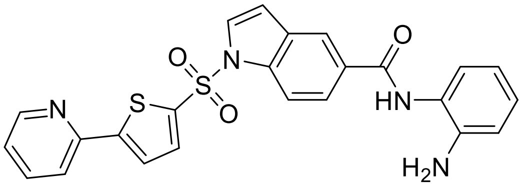	474.55	3.90
**MC1742**	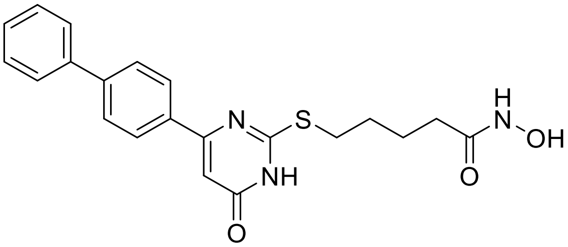	395.48	2.64
**MC1862**	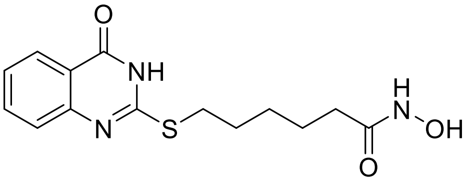	307.37	0.78

**MC2126**	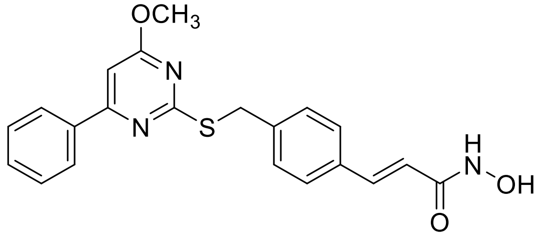	393.46	4.10
**MC2129**	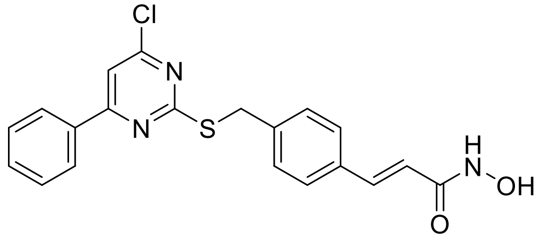	397.88	3.90
**JMF-1080**	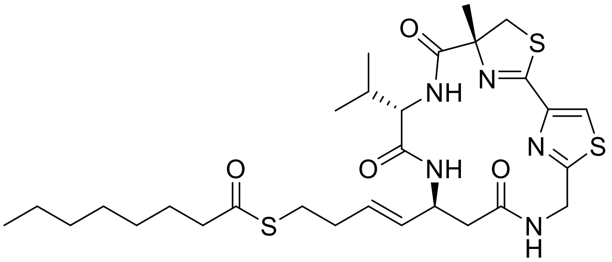	621.87	4.84
**SD-L-256**	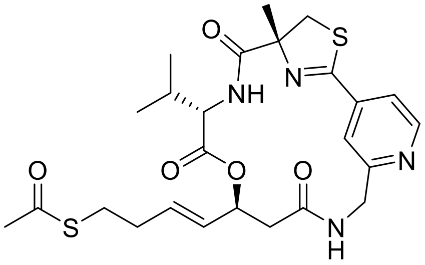	532.67	1.92
**SD-L-148 (Largarole)**	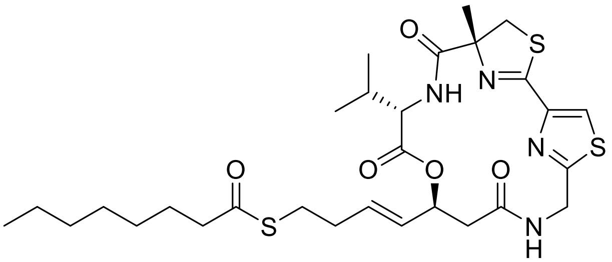	622.86	4.93
**GRM1 (SAHA)**	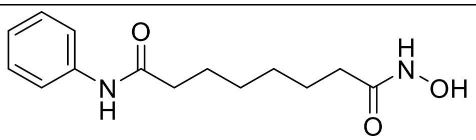	264.32	0.99
**GRM2 (tubastatin)**	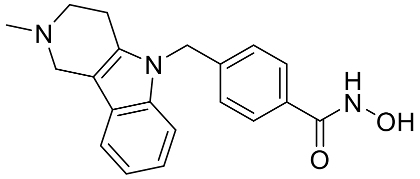	335.41	2.13

**GRM3 (entinostat)**	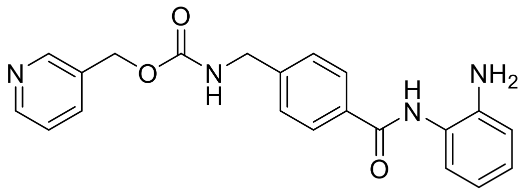	376.42	-0.12
